# Temperature tolerance and persistence of promising native *Metarhizium* isolates for use in chili pepper farms in Ghana

**DOI:** 10.3389/ffunb.2026.1780941

**Published:** 2026-04-14

**Authors:** Patricia Akua Sitsofe Nyahe, Vincent Yao Eziah, Candice Anne Coombes, Samuel Kumahor, Isaac Nortey, Monica Akumyoungta, Drauzio E. N. Rangel, Alene Alder-Rangel, Michael Yao Osae, Dalia Sukmawati, Mavis Agyeiwaa Acheampong

**Affiliations:** 1 Department of Crop Science, University of Ghana, Accra, Ghana; 2 Centre for Biological Control (CBC), Department of Zoology and Entomology, Rhodes University, Makhanda, South Africa; 3 Department of Soil Science, University of Ghana, Accra, Ghana; 4 Inbioter Institute of Biotechnology Rangel, Itatiba, Brazil; 5 Instituto de Ciência e Tecnologia, Universidade Federal de São Paulo,São José dos, Campos, Brazil; 6 Alder’s English Services, São José dos Campos, Brazil; 7 Biotechnology and Nuclear Agriculture Research Institute (BNARI), Ghana Atomic Energy Commission, Accra, Ghana; 8 Department of Biology, Faculty of Mathematics and Natural Sciences, Universitas Negeri Jakarta, Rawamangun, Indonesia

**Keywords:** chili pepper, entomopathogenic fungi, false codling moth, *Metarhizium* spp., persistence, temperature tolerance

## Abstract

Ghanaian chili pepper exports are subject to stringent regulations on pesticide residues in significant foreign markets. Therefore, safer pest management options are needed to supplement current non-chemical control techniques, especially for controlling *Thaumatotibia leucotreta* Meyrick (False codling moth, FCM), an important phytosanitary pest. A national survey was initiated in 2023 to identify native entomopathogenic fungi (EPF) from Ghanaian farms. Seven *Metarhizium* isolates were recovered from soils and induced substantial mortalities of soil-residing final-instar stages of FCM *in vitro*. To advance the development of a mycoinsecticide targeting FCM and other key insect pests of chili pepper, the present study evaluated the *in vitro* temperature tolerance of the seven isolates and persistence of two (UGSUHC1 and UGJKCS9) under field conditions. Temperature tolerance assays based on *in vitro* radial growth (at 6–40 °C) indicated that all isolates could grow between 15 and 35 °C, with optimum development occurring between 25 and 27 °C. No growth occurred at 6, 8, 38, and 40 °C. Two-month semi-field persistence trials using sterile soil inoculated with each respective isolate and buried in chili pepper farms at Adidome, Peki, and the University of Ghana, demonstrated that both isolates remained viable in the soil throughout the trial period and retained their infectivity against FCM. These results highlight the potential of these Ghanaian isolates against subterranean life stages of FCM. The need for further evaluations, particularly field trials, is therefore imperative for the development of an effective and sustainable control option for FCM in chili peppers in Ghana.

## 1 Introduction

False codling moth (FCM), *Thaumatotibia leucotreta* Meyrick (Lepidoptera: Tortricidae), is a major constraint to chili pepper exports from Ghana because the EU classifies it as a quarantine pest (EUROPHYT (European Union Notification System for Plant Health Interceptions), 2014; Fening et al., 2020). Locally, FCM also causes severe yield losses. The larvae of FCM bore into fruits, inducing premature ripening, decay, and fruit drop (Boardman et al., 2012; Adom et al., 2023, 2024). Repeated interceptions of infested consignments led to an EU ban on Ghana’s chili pepper exports between 2015 and 2017, resulting in an estimated revenue loss of approximately USD 30 million (Fening et al., 2020). Consequently, Ghana’s chili pepper export volumes and earnings declined sharply, from 984 to 1,080 metric tons (US$ 350,000–1.18 million) in 2010–2014 to only US$ 351,000–87,000 between 2018 and 2021 (GEPA (Ghana Export Promotion Authority), 2021; GIRSAL (Ghana Incentive-Based Risk Sharing System. for Agricultural Lending), 2023).

Synthetic pesticides, which remain the primary control strategy for FCM in Ghana, have not achieved adequate control due to a limited lethal window against the unnoticeable eggs and first instars on fruits (Fening et al., 2017; Mbatchou et al., 2024). Additionally, conventional pesticides face stringent regulation in export markets and pose risks to the environment, non-target organisms, and human health (Pathak et al., 2022; Asamani et al., 2024; Peprah et al., 2025; Tachie-Menson et al., 2025; Wan et al., 2025). These challenges highlight the urgent need for effective and sustainable non-chemical alternatives for FCM management.

Seven native *Metarhizium* isolates (UGJKCS9, UGAFMF8, UGSUHCI, UGJKCS10, UGNAKC1, UGKAP1, and UGAFMF12) recovered from agricultural soils in Ghana have exhibited strong pathogenicity against the pre-pupating stages of FCM (Ameyaw et al., 2026). All isolates induced more than 80% pupal mortality with low LC_50_ and LT_50_ values of 2.7–9.9 × 10^6^ conidia/mL and three days, respectively (Ameyaw et al., 2026). Although all seven *Metarhizium* isolates were found to be highly sensitive to UV radiation (Nyahe et al., 2025), the thermal limits of these isolates remain unknown. Like UV radiation, temperature is an equally important efficacy parameter regulating the growth, infectivity, survival, and persistence of entomopathogenic fungi (EPF) (Jaronski, 2010; Acheampong et al., 2020b; Quesada-Moraga et al., 2024). Most isolates exhibit optimal growth and virulence between 20 and 30 °C, and an upper temperature limit between 35 and 40 °C, with appreciable decline in performance outside this range, although thresholds vary among isolates (Yeo et al., 2003; Dimbi et al., 2004; Rangel et al., 2005; Acheampong et al., 2020b; Velavan et al., 2022; Chailleux et al., 2023; Sui et al., 2025). While UV radiation and temperature may not be the most detrimental environmental factors impacting these isolates when applied against soil-inhabiting stages of FCM, they will be more consequential when used against above-ground pests of pepper. As the intended EPF-based product will be used to control both above- and below-ground stages of FCM, as well as other foliar pests of chili pepper (notably thrips, aphids, whiteflies, and fruit flies), selecting isolates with UV and thermal tolerances that align with Ghanaian field conditions is essential to ensure consistent and reliable field performance.

Another crucial issue that must be addressed towards the development of a mycoinsecticide product from these seven *Metarhizium* isolates for FCM control is their persistence in the various agroecological conditions in Ghana. Soil is the main natural habitat of EPF, enabling them to survive in this niche for six months (Coombes et al., 2013, 2016; Yang et al., 2019) or one to three years (Vänninen et al., 2000; Milner et al., 2003; Pilz et al., 2011; Yang et al., 2019). However, their persistence in augmentative biological control is highly dependent on the isolate, applied environment, soil factors (e.g., type of soil and moisture levels), other microbes, and abiotic factors, among others (Jaronski et al., 2007 , 2010; Lacey et al., 2015). It is not known how these isolates would perform under field conditions in Ghana as this is the first EPF study on chilli pepper in Ghana. Knowing whether the seven Ghanaian *Metarhizium* isolates can tolerate the tropical field conditions in Ghana and persist long enough to control FCM in chili pepper farms is critical for their development as bioinsecticides. Thus, this study aims to determine the temperature tolerances of all seven promising native *Metarhizium* isolates under laboratory conditions and evaluate the persistence of the two most promising isolates in chili pepper farms in Ghana. The findings from this study will be factored into the selection of the most suitable isolate for future development into a mycoinsecticide product for the chili pepper environment in Ghana. The development of such a product will reduce overreliance on synthetic pesticides and complement non-chemical control strategies being developed against FCM in Ghana, permitting increased production to meet export demand.

## 2 Materials and methods

### 2.1 Source of insects and fungal isolates

Final-instar larvae of FCM were obtained from the African Regional Postgraduate Programme in Insect Science (ARPPIS), University of Ghana, where cultures for research are maintained on an artificial larval diet (Moore et al., 2014). The seven Ghanaian *Metarhizium* isolates (UGSUHC1, UGJKCS9, UGJKCS10, UGKAP1, UGNAKC1, UGAFMF8, UGAFMF12) were sourced from the Entomopathology Laboratory at ARPPIS, where their conidia are preserved on Sabouraud dextrose agar (SDA) slants at 4 °C. These isolates were originally obtained from soils collected from chili pepper, maize, and cocoa farms in the Central, Eastern, and Greater Accra Regions of Ghana using *Galleria mellonella* (Lepidoptera: Pyralidae) as the bait insect (Goble et al., 2010). The geographical origin, molecular identification tool and GenBank accession numbers of all seven isolates were provided in Nyahe et al. (2025). All isolates were passed once through fifth-instar FCM larvae before use, following Acheampong et al. (2020a). Conidia harvested from infected cadavers were cultured on SDA at 25 °C and stored at 4 °C as stock cultures for subsequent assays.

### 2.2 Effect of temperature on radial mycelial growth of the seven *Metarhizium* isolates

#### 2.2.1 Temperature tolerance protocol

For each isolate, a 6 mm mycelial plug from three-day-old cultures (from subcultures on SDA for 14 days at 27 °C, 60% RH, with a 12 h photoperiod) was placed in the center of an SDA Petri dish (90 mm), with five replicates per isolate. The dishes were then placed in a transparent plastic box (18 × 27 × 18 cm), and each box, containing five replicates of each isolate, was incubated for 15 days in the dark at eight tested temperatures (6, 8, 15, 20, 25, 35, 38, and 40 ± 1 °C). Radial growth measurements were recorded every two days starting from the third day, along four transects, using two perpendicular lines at the back of each plate (Acheampong et al., 2020b). The entire experiment was performed twice with new suspensions from each isolate.

### 2.3 Persistence protocol

The persistence of two out of the seven *Metarhizium* isolates (UGJKCS9 and UGSUHC1), which caused higher mycosis of soil-residing stages of FCM in previous virulence assays (Ameyaw et al., 2026), was evaluated in three chili pepper farms located in Adidome (6°06′22.50″ N, 0°30′26.39″ E), Peki (6°33′5.11″ N, 0°15′17.35″ E), and the University of Ghana (5°39′34.16″ N, 0°11′37.68″ W), following the protocol of Coombes et al. (2013) with minor modifications.

Isolates were subcultured on SDA at 27 °C, 60% RH, with a 12 h photoperiod for two weeks to obtain conidia. Conidia were mass produced on rice medium following the protocol of Sahayaraj and Namasivayam (2008). For each isolate, a 100 g sterile (autoclaved three times at 121 °C for 15 min) soil sample collected from the respective chili pepper farms where the study was undertaken was combined with 0.5 g of fungal-colonized rice grains and kept in a mesh bag (60 × 40 mm size, 0.25–0.4 mm mesh) at four replicates. Four bags per isolate were used as the initial (time-zero) counts of their colony-forming units (CFU) per gram of soil (CFU/g). The soil-inoculated bags were buried under chili pepper plants (just below the soil surface [~5 cm depth], where final-instar larvae of FCM pupate). Non-inoculated control bags containing only sterile soil were also buried to determine whether any potentially competing fungi could relocate into the sterile soil. The persistence was done in sterile soils to isolate the effect of the fungus itself without interference from other competing microbes.

Four replicate bags of each isolate were transported to the laboratory every two weeks for 8 weeks (July and August 2025) to determine the CFU/g. In the laboratory, each bag was thoroughly mixed, and 1 g of soil from a 50 g sample was suspended in 1 mL of 1% Tween 20 solution and vortexed. Serial dilutions were done, and 100 µL aliquots were spread-plated on three replicate SDA dishes amended with 50 mg/L each of streptomycin and chloramphenicol to inhibit potential bacterial growth. Plates were incubated at 25 °C for five to ten days, after which the CFU/g were counted. Morphocultural confirmation of the inoculated fungal species was performed, and non-target colonies were excluded from the count.

In addition to the CFU counts, an assay was undertaken to determine whether the conidia in each sample were capable of inducing mortality of FCM final-instar larvae. For each isolate, the remaining 50 g of soil from each collected bag was placed individually in a 90 mm sterile Petri dish, and 10 final-instar larvae of FCM were added. Each Petri dish was then covered with an inverted 2 L transparent plastic cup cut at the top with a cotton-plugged hole moistened with sterile water, which served as an adult emergence chamber for the pre-pupating larvae. The treatments were maintained under ambient conditions in the laboratory (27 °C, 60% RH, with a 12 h photoperiod), and the assay was terminated 10 days after first adult emergence (Coombes et al., 2013). The number of live and dead larvae, pupae, and adults was recorded daily. Most cadavers in fungal-treated soils sporulated within seven days. For those that did not sporulate in the soil, death due to mycosis was verified by surface sterilizing cadavers in 0.5% sodium hypochlorite (3.5%) and 70% ethanol (2 min each), followed by seven days of incubation on sterile moistened filter paper in Petri dishes at the same ambient conditions above.

### 2.4 Statistical analyses

The *Metarhizium* isolates grew linearly, fitting the linear model, y = vt + b, where the slope (v) represents the growth rate (mm/day) at each incubation temperature (t) (Ouedraogo et al., 1997). Therefore, the data for each isolate at each temperature were subjected to a linear regression analysis. The growth rates from the regression analysis were fitted to a generalized beta function below to estimate the minimum, maximum, and optimum growth temperatures of each isolate (Bassanezi et al., 1998; Quesada-Moraga et al., 2006; García-Fernández et al., 2008; Acheampong et al., 2020b; Omuse et al., 2022).

The generalized β function is as follows:


$$Y(T)=TY_{opt}{\left[(T-T_{min})/(T_{opt}-T_{min})\right]}^{TB_{3}(T_{opt}-T_{min})/(T_{max}-T_{opt})}\times (T_{max}-T)/{\left(T_{max}-T_{opt}\right)}^{TB_{3}}$$

Where,

Y(T) = fungal growth rate (mm/day) (dependent variable); T = incubation temperature (independent variable); Tmin, Tmax, and Topt represent the lowest, highest, and optimum temperature of the fungus, respectively; TYopt = rate of growth at the optimum temperature (Topt); and the shape parameter, TB_3_ = the range of temperatures around Topt where growth is near TYopt. When TB_3_ values are low (e.g., 0.1), growth is maintained over a wide temperature range, and high values (e.g., 3.0) suggest a steep drop in growth when temperature is changed slightly outside the range of Topt (Bassanezi et al., 1998). The minimum temperature (Tmin) was fixed at 8 °C, as no radial growth occurred for any isolate at 6 °C and 8 °C. This modification enhanced the model’s fit and reduced the standard errors of the parameter estimates.

TYopt, Topt, Tmax, and TB_3_ and their standard errors were estimated via Levenberg-Marquardt non-linear least squares algorithm (Bates and Watts, 1988) with the “nlsLM” function of the ‘minipack.lm’ package. Parameter estimates were contrasted using pairwise t-tests adjusted with Bonferroni correction to address type I errors.

No EPF was recorded in the non-inoculated control soil bags buried at all study sites. Therefore, they were excluded from the CFU count analyses. The CFU data that were not normally distributed even after transformations were subjected to a generalized linear model (GLM) with gamma error distribution, which yielded optimal goodness of fit (lowest Akaike Information Criteria value and Likelihood Ratio Test) (Acheampong et al., 2020a). The cumulative mortality data (larvae, pupae, and adults) were not corrected for control mortality, as death due to natural occurrences was less than 5%. The mortality data were also subjected to GLM (family = binomial). Analysis of deviance (ANODEV) was applied to the GLM models and contrasted using *emmeans* (Lenth, 2024) with Tukey HSD test (α = 0.05). All analyses were done in R version 4.4.2 (R Core Team, 2024).

## 3 Results

### 3.1 Effects of temperature on fungal isolates

**Figure 1:**
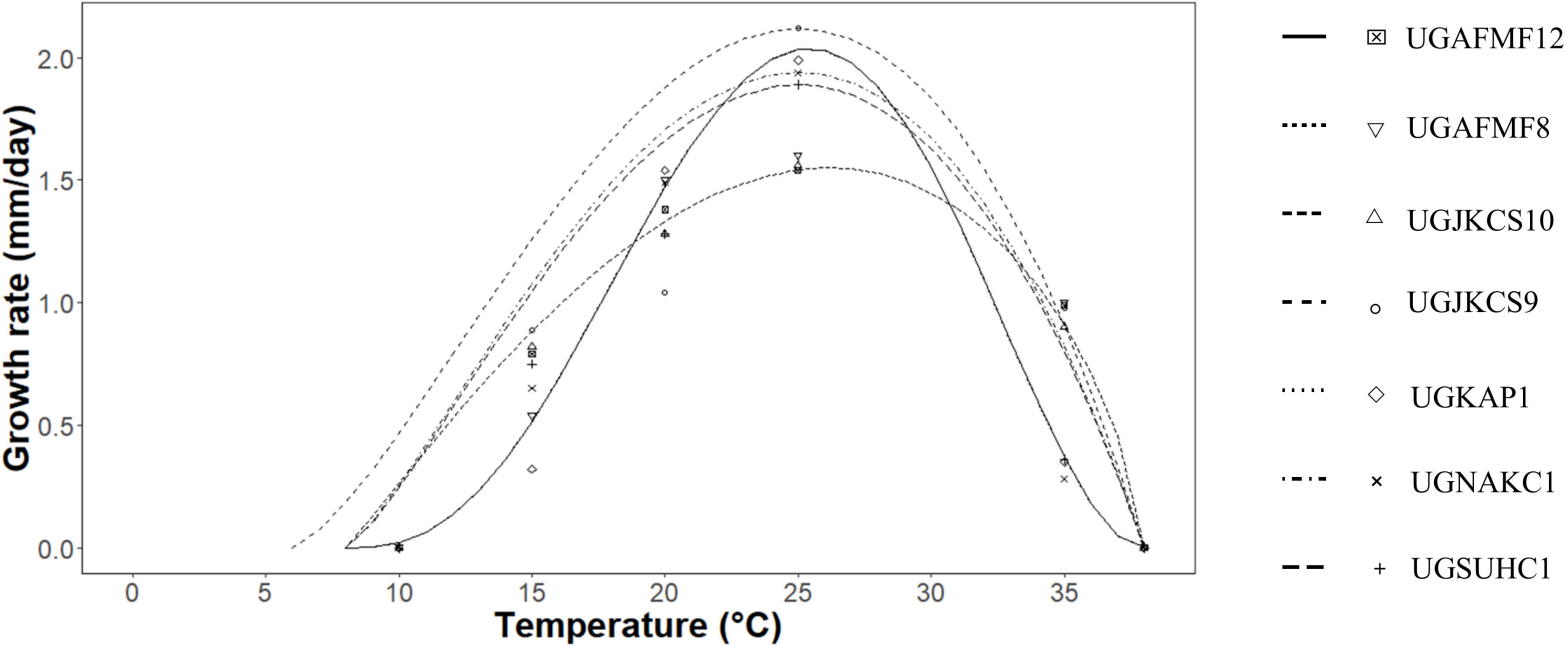
Effect of temperature on radial growth of the seven *Metarhizium* isolates. The modified β function (Bassanezi et al., 1998) was fitted to the average growth rates (mm/day) of fungal isolates at the different temperatures tested to estimate minimum, maximum, and optimal temperatures for the isolates.

All seven Ghanaian *Metarhizium* isolates exhibited similar thermal limits, growing between 15 and 35 °C with no growth occurring at 6, 8, 38, and 40 °C. The optimum temperatures for growth (Topt) ranged between 25 and 27 °C, which did not differ statistically among the isolates (**Table 1**). The highest growth rate at the optimum temperature (TYopt) occurred for UGKAP1 (2.0 mm/day), although it did not differ significantly from those recorded for UGJKCS9 (1.9 mm/day), UGNAKC1 (1.9 mm/day), and UGSUHC1 (1.8 mm/day). The maximum growth temperature (Tmax) for the isolates ranged from 37.2 to 38.8 °C. The shape parameter (TB_3_) ranged from 0.8 to 2.2, showing low to high values for the tested isolates. The modified β function was used to accurately represent fungal growth rate-temperature curves in the study (**Figure 1**).

**Table 1:** Parameters estimated (± SE) from the β function^1^ fitted to the radial growth data of the seven Ghanaian *Metarhizium* isolates.

Isolate	Estimated Parameters^6^			
	TB_3_ (°C)^2^	TYopt (mm/day)^3^	Tmax (°C)^4^	Topt (°C)^5^
UGJKCS9	0.9 (0.0) b	1.9 (0.1) ab	38.1 (0.1) a	25.9 (0.4) a
UGJKCS10	0.8 (0.1) b	1.5 (0.1) c	38.1 (0.1) a	25.8 (0.3) a
UGSUHCI	1.1 (0.1) b	1.8 (0.1) ab	38.0 (0.1) a	24.7 (0.9) a
UGNAKC1	1.8 (0.3) a	1.9 (0.1) ab	37.2 (1.6) a	25.8 (1.4) a
UGKAP1	2.2 (0.3) a	2.0 (0.2) a	38.8 (0.5) a	25.6 (0.4) a
UGAFMF8	0.8 (0.0) b	1.7 (0.0) bc	38.0 (0.0) a	27.0 (0.2) a
UGAFMF12	0.8 (0.0) b	1.7 (0.0) bc	38.0 (0.0) a	27.0 (0.2) a

^1^modified β function given as Y(T) = TYopt/((T-Tmin)/(Topt-Tmin)) ^ (TB_3_* ((Topt-Tmin)/(Tmax-Topt))) *((Tmax-T)/(Tmax-Topt)) ^TB_3_, Y(T) is the fungal growth in mm/day, and T is the incubation temperature (Bassanezi et al., 1998). Tmin is the minimum temperature for fungal growth, fixed at 8 °C.

^2^ TB_3_ is defined as the shape parameter that influences the temperature range around Topt, where the curve stays near TYopt.

^3^ TYopt is the fungal growth at the optimal temperature.

^4^ Tmax is the maximum temperature for fungal growth.

^5^ Topt is the optimal temperature for fungal growth.

^6^ Means within a column with a different letter differ statistically (Pairwise t-test, P < 0.05 with Bonferroni correction).

### 3.2 Persistence of Ghanaian *Metarhizium* isolates in chili pepper farms

The three-way ANODEV revealed that assessment week (χ^2^ = 8123.20, df = 4, P < 0.001) was the only main effect that significantly influenced the CFU count. The fungal isolate (χ^2^ = 0.00, df = 1, P = 1.000), and location (χ^2^ = 0.00, df = 2, P = 1.000) were not significant. However, all associated interactions of the three factors [fungal isolate × field location (χ^2^ = 27.80, df = 2, P < 0.001), location × assessment week (χ^2^ = 71.90, df = 8, P < 0.001), fungal isolate × assessment week (χ^2^ = 173.30, df = 4, P < 0.001), and fungal isolate × location × assessment week (χ^2^ = 72.10, df = 8, P < 0.001)] were significant.

**Figure 2:**
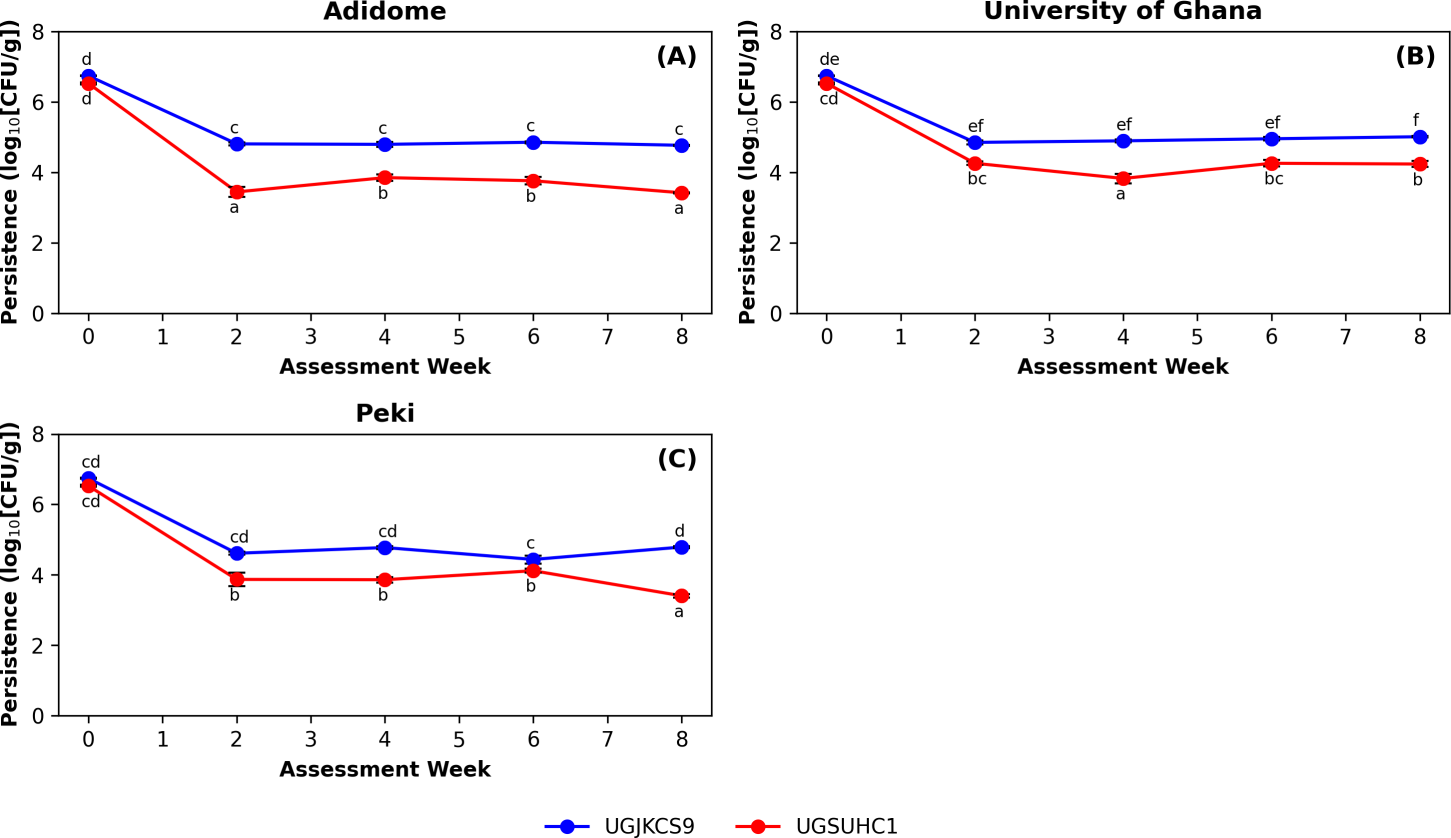
Bimonthly CFU counts of the two Ghanaian *Metarhizium* isolates (UGJKCS9 and UGSUHC1) over the two months at Adidome **(A)**, University of Ghana **(B)** and Peki **(C)**. Blocks represent the means, and the bars are the standard errors of four replicates. Means with the same lowercase letter did not differ significantly (‘emmeans’ adjusted with Tukey’s HSD test, P > 0.05) for both within-week and between-week comparisons.

The initial concentration of UGJKCS9 (5.5 × 10^6^ CFU/g of soil) and UGSUHC1 (3.4 × 10^6^ CFU/g of soil) declined significantly across all tested sites after two weeks of burial. At Adidome (**Figure 2A**), the week of assessment (χ^2^ = 10567, df = 4, P < 0.001) and its interaction with fungal isolate (χ^2^ = 110.00, df = 4, P < 0.001) significantly affected CFU counts. However, the fungal isolate (χ^2^ = 0.00, df = 1, P = 1.000) was not significant. UGJKCS9 maintained a steady persistence after the initial decline until the end of the assessment period. On the other hand, UGSUHC1 increased after the decline at week four and maintained a steady increase until it reached a count statistically similar to week two.

At the University of Ghana (**Figure 2B**), the fungal isolate (χ^2^ = 39.28, df = 1, P < 0.001), assessment week (χ^2^ = 2495.76, df = 4, P < 0.001), and their interaction (χ^2^ = 52.55, df = 4, P < 0.001) were significant. UGJKCS9 exhibited an initial decline in CFU at week two, maintaining a constant count until week eight. UGSUHC1 displayed a similar decline in concentration at week two, reducing further at week four, before increasing at week six. The CFU count at week eight did not differ from that recorded at week two.

At Peki (**Figure 2C**), a significant interaction between fungal isolate and week of assessment (χ^2^ = 86.50, df = 4, P < 0.001) was noted. Assessment week was the only significant main effect (χ^2^ = 2899.90, df = 4, P < 0.001). Fungal isolate was not significant (χ^2^ = 0.00, df = 1, P = 1.000). UGJKCS9 recorded a drastic decline at week two, which increased at week four, decreased at week six, and then increased to a statistically similar count as in week two. The CFU count of UGSUHC1 decreased at week two, increasing steadily from week four to six before a drastic decline to a significantly lower count compared to the other assessment weeks.

### 3.3 Virulence of Ghanaian *Metarhizium* isolates following burial in chili pepper farms

Three-way ANODEV showed that only treatment (χ^2^ = 1731.63, df = 2, P < 0.001) significantly influenced cumulative mortality. The location (χ^2^ = 2.14, df = 2, P = 0.344), assessment week (χ^2^ = 6.26, df = 4, P = 0.181), and associated interactions [treatment × location (χ^2^ = 1.56, df = 4, P = 0.816), treatment × assessment week (χ^2^ = 6.91, df = 8, P = 0.546), location × assessment week (χ^2^ = 2.96, df = 8, P = 0.937), and treatment × location × assessment week (χ^2^ = 3.09, df = 16, P = 0.999)] were not significant.

**Figure 3:**
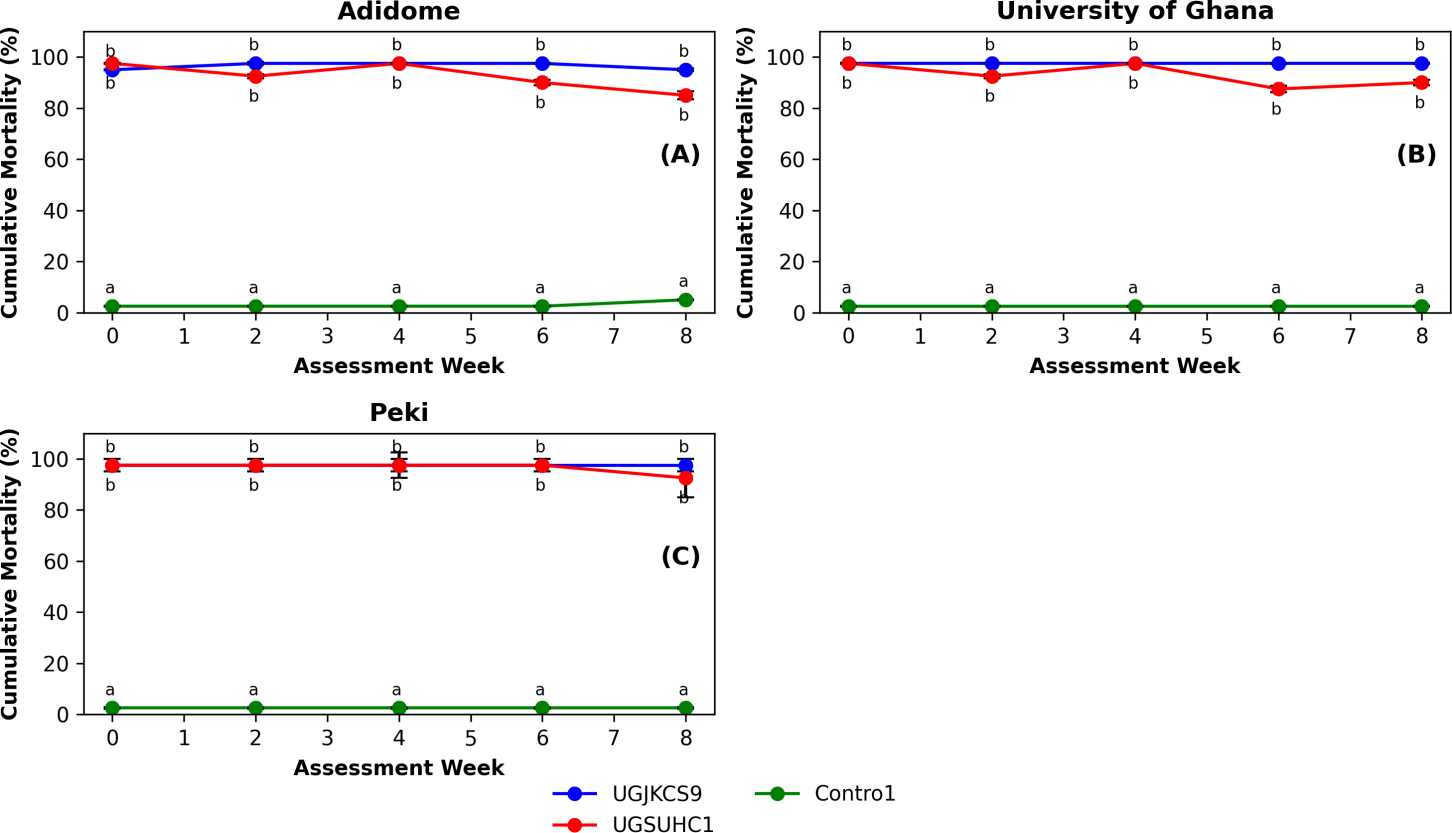
Bimonthly percentage mortality of the two Ghanaian *Metarhizium* isolates (UGJKCS9 and UGSUHC1) over the two months at Adidome **(A)**, University of Ghana **(B)** and Peki **(C)**. Blocks represent the means, and the whiskers are the standard errors of four replicates. Means with the same lowercase letter did not differ significantly (‘emmeans’ adjusted with Tukey’s HSD test, P > 0.05) for both within-week and between-week comparisons.

At each assessment period, both fungal isolates caused over 85% mortality (**Figures 3A–C**). The mortalities induced by these isolates did not differ from one another across the assessment periods. The cumulative mortalities in the non-inoculated control treatments remained below 5%. The percentage of mycosis in the fungal treatments exceeded 90% for all the fungal isolates (data not presented). No mycosis was observed on the non-inoculated controls at each assessment week.

#### 3.3.1 Weather data at experimental sites

**Figure 4:**
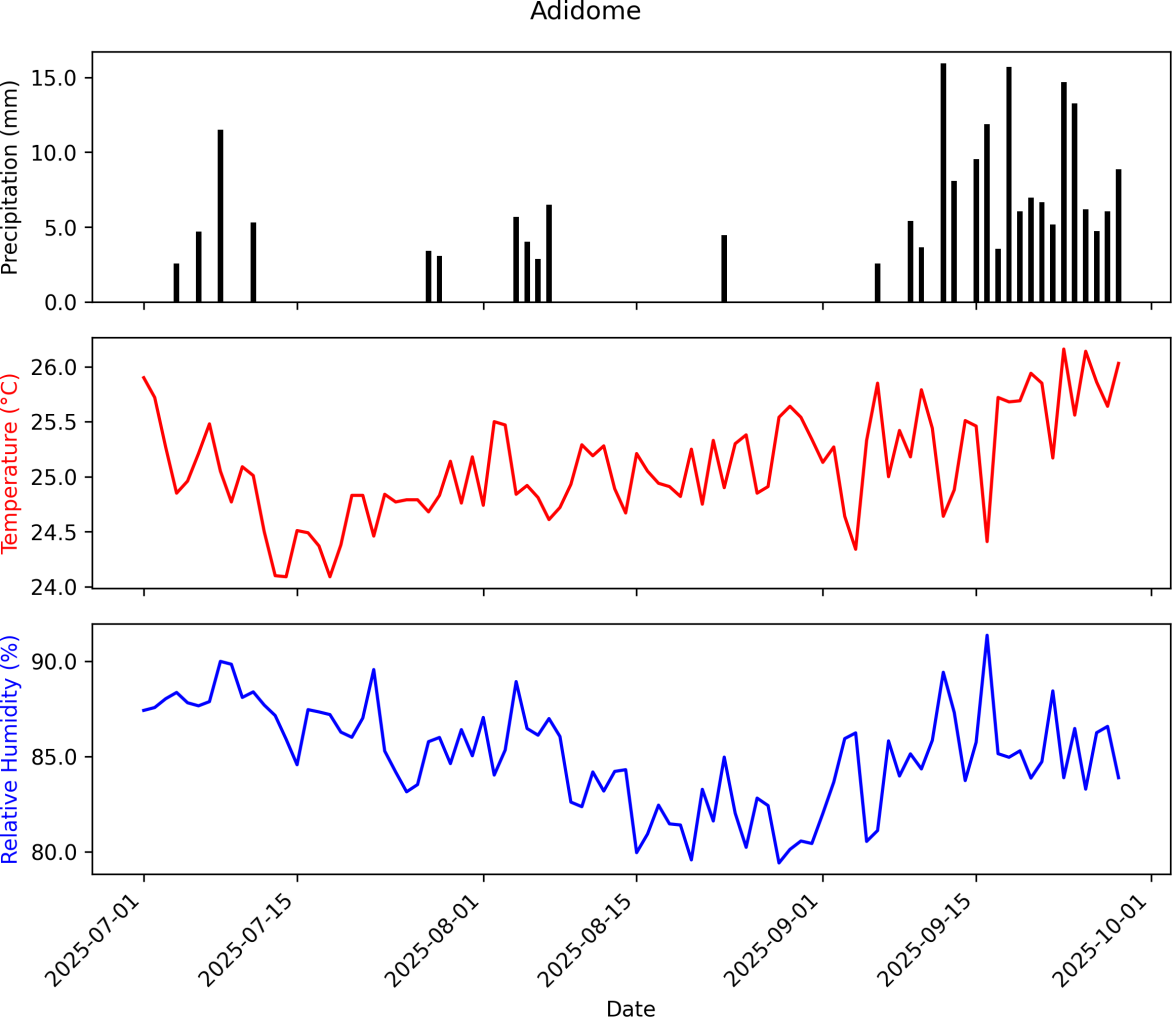
Summary of weather data recorded from July 1 to September 28, 2025, at Adidome: Mean temperature = 25.10 °C, Mean Relative Humidity = 85.02%, Cumulative Precipitation = 209.12 mm.

**Figure 5:**
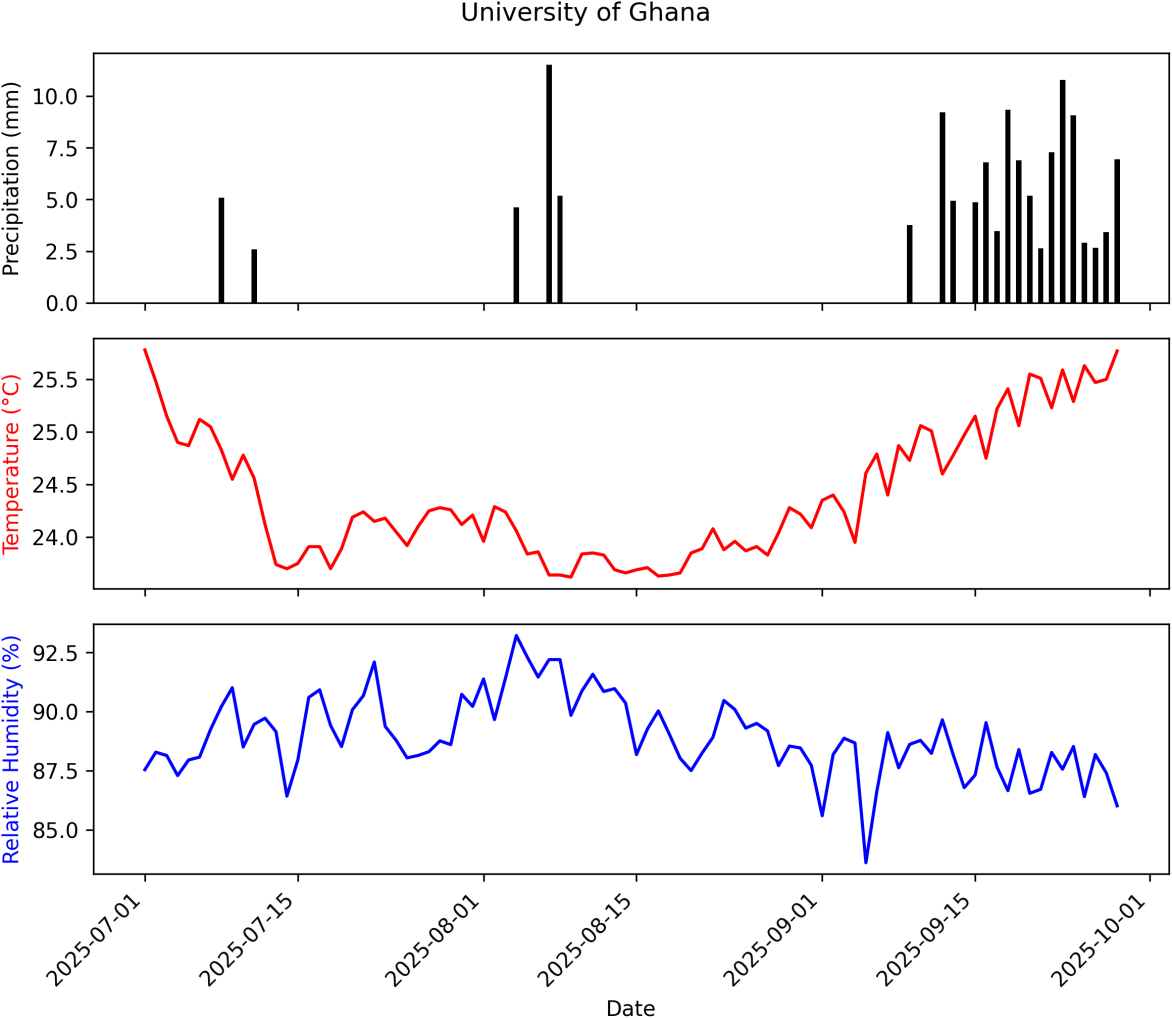
Summary of weather data recorded from July 1 to September 28, 2025, at University of Ghana: Temperature = 24.40 °C, Relative Humidity = 88.94%, Cumulative Precipitation = 129.00 mm.

**Figure 6:**
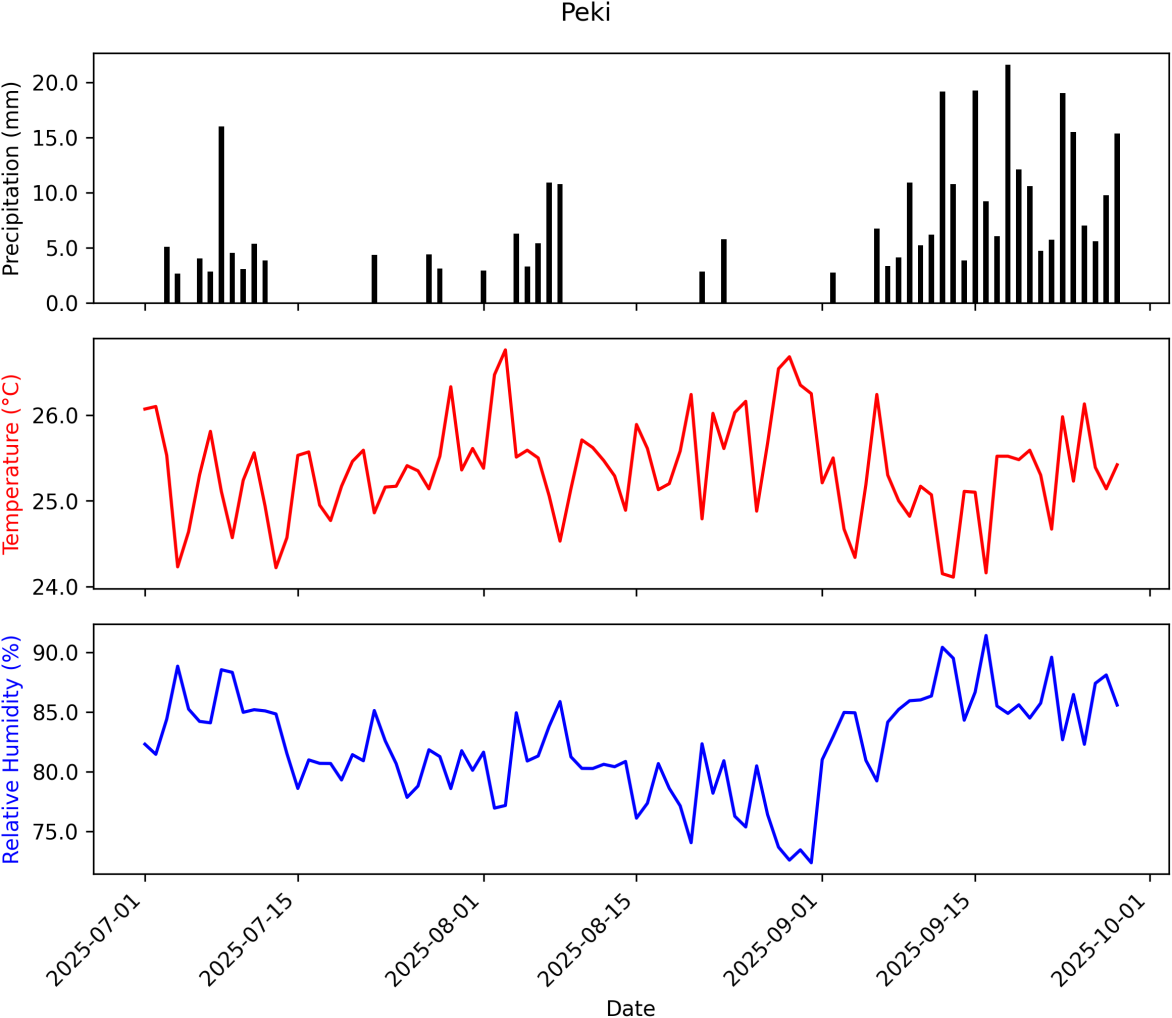
Summary of weather data recorded from July 1 to September 28, 2025, at Peki: Temperature = 25.37 °C, Relative Humidity = 82.16%, Cumulative Precipitation = 342.04 mm.

The daily temperature, relative humidity, and precipitation data for the EPF persistence trials were obtained from NASA POWER database. The mean monthly temperature, relative humidity, and cumulative precipitation were 25 °C, 85% and 209 mm at Adidome (**Figure 4**); 24 °C, 89%, and 129 mm at University of Ghana (**Figure 5**); and 25 °C, 82%, and 342 mm at Peki (**Figure 6**) (NASA POWER, 2025).

## 4 Discussion

Abiotic environmental competence is a key factor in isolate selection for mycoinsecticide product development (Fernandes et al., 2015; Tumuhaise et al., 2018; Acheampong et al., 2020a). This is important because abiotic environmental factors, especially temperature, UV radiation, and humidity, influence the virulence and subsequent survival and persistence in the applied environment (Jaronski, 2010; Dias et al., 2018; Rangel and Roberts, 2018, 2023; Quesada-Moraga et al., 2024). Therefore, this study addressed the temperature tolerance and persistence of virulent Ghanaian *Metarhizium* isolates being developed to control key pests of chili pepper in Ghana, especially FCM.

The seven Ghanaian *Metarhizium* isolates exhibited similar thermal limits, growing between 15 and 35 °C, with optimal growth at 25–27 °C. The optimum temperatures fall well within the daily temperature brackets at the three chilli pepper farms in this study (**Figures 4**–**6**), meaning that the isolates are likely to perform effectively under natural conditions. These results strengthen confidence in the ecological relevance of the fitted model and further underscore the potential resilience of the isolates to climatic variability, as their peak growth performance coincides with the prevailing thermal regime.

The mesophilic growth of these tropical *Metarhizium* isolates corroborates the extensive earlier findings of these filamentous biocontrol agents (Ekesi et al., 1999; Bidochka et al., 2002; Rangel et al., 2005; Bugeme et al., 2008; Chandra Teja and Rahman, 2016; Fernández-Bravo et al., 2016; Tumuhaise et al., 2018; Quesada-Moraga et al., 2024). The rapid growth of EPF is advantageous for effective pathogenesis, especially under the continually changing environmental conditions (Goettel and Inglis, 1997; Inglis et al., 2001), and for improving efficiency during mass production (Jaronski, 2013). However, the thermal limits are often more important for persistence, regulating survival outside their insect hosts under field conditions. The modified beta function predicted 38 °C as the maximum thermal limit for the majority of the isolates, suggesting that they could survive in hotter (daytime highs > 35 °C) chili pepper growing regions in Ghana, particularly the Northern sector. It is also envisaged that more optimum than lethal temperatures will prevail in the field, with soil temperatures likely cooler and more stable for the applied EPF in a formulation targeting the soil environment. Prolonged exposure to high temperatures may, however, be detrimental for formulations targeting the foliar arthropod pests of chilli pepper.

The two *Metarhizium* isolates tested persisted in the field for two months. They were still able to induce more than 80% mortality of FCM final-instar larvae *in vitro*, which aligns with previous studies with *Metarhizium* spp. targeting soil-dwelling stages of the same pest (Coombes et al., 2013, 2016) and other pests (Ekesi et al., 2005; Vidal and Kuhlmann, 2005; Ekesi et al., 2011; Yang et al., 2019). While *Metarhizium* spp. are particularly adapted to varying environmental conditions (Quesada-Moraga et al., 2007; Vänninen et al., 2000; Chen et al., 2014; St. Leger and Wang, 2020; Stone and Bidochka, 2020; Fernández-Bravo et al., 2021), the marked reduction in fungal concentration two weeks after burial is not uncommon. Similar rapid initial reductions in concentration followed by gradual steady decline in EPF field efficacy or persistence studies are established (Rath et al., 1995; Shimazu et al., 2002; Milner et al., 2003; Scheepmaker and Butt, 2010; Ekesi et al., 2011; Coombes et al., 2013; Yang et al., 2019). Interestingly, a 2 to 10-fold increase in baseline concentration of the applied EPF, which was attributed to increase in the infestations of target hosts (enhancing recycling), has also been reported (Rath et al., 1995; Kessler et al., 2004). Likewise, approximately a 17-fold increase in the initial concentration of granular formulations of *B. bassiana* and *M. anisopliae* s.l. isolates after two weeks in treated plots was attributed to proliferation on its nutritious substrate (millet) (Parker et al., 2015).

The fungal persistence proffered in this study is based on performance in sterile soil. Many studies have found that EPF persistence in sterile soil is significantly greater than in non-sterile soil due to exploitative and interference competition with other microbes and invertebrate decomposers (Lingg and Donaldson, 1981; Keller et al., 2003; Kessler et al., 2004; Scheepmaker and Butt, 2010; Coombes et al., 2013). Additionally, the field persistence of EPF is regulated by the type of formulation (Inyang et al., 2000; Ekesi et al., 2005) and abiotic factors (temperature, UV radiation, moisture, rainfall, soil type, organic matter content, etc.) (Butt, 2002; Keller et al., 2003; Rumbos et al., 2008; Garrido-Jurado et al., 2011), among others (Butt et al., 2001; Keller et al., 2003; Zimmermann, 2007). Therefore, the current results cannot be fully generalized to the actual persistence in the field. However, environmental conditions obtained from the three field sites (Mean air temperatures = ~25 °C, Relative humidity = 85%, Cumulative precipitation = 218 mm) match the optimal conditions for EPF growth, conidiation, and persistence (Quesada-Moraga et al., 2024; Acheampong et al., 2020b). It is also interesting to note that although there was this initial decline in fungal concentration, virulence was still retained, which corroborates the previous laboratory virulence of these isolates (Ameyaw et al., 2026). This sustained virulence may also ensure that the fungus continues to recycle within the host environment, providing prolonged control. Furthermore, the two isolates used for the field trials have shown promising efficacy against the oriental fruit fly (*Bactrocera dorsalis* Hendel) (Diptera: Tephritidae) (Amissah, 2025), which is an equally important phytosanitary pest on chili pepper. Like *Beauveria* spp., *Metarhizium* spp. have a wider host range, and could potentially infect other chili pepper pests and other non-targeted hosts to further enhance conidial recycling and concentrations in the field. Thus, a mycoinsecticide formulated from these isolates has the potential to persist for at least two months and control FCM in chili pepper farms in Ghana. A granular formulation on a nutritive substrate applied in the soil environment, that is far more stable than above ground would be ideal for persistence and control of soil-dwelling stages of FCM. However, a product suitable for controlling both above and below-ground pests is required, and therefore a more suitable formulation that also affords a level of UV protection for use in foliar pest control in Ghanaian chili peppers will need to be developed.

## 5 Conclusions

This manuscript provides evidence to support the thermal competence and a minimum of two months viable semi-field persistence for these highly virulent *Metarhizium* spp. isolates in Ghanaian chili soils, targeting the key pest, FCM. While further research is required to definitively conclude the outcomes of this study in a field setting, these results represent a positive step forward in the development of a more sustainable and environmentally conscious biopesticide for controlling important insect pests in Ghana.

## Data Availability

The raw data supporting the conclusions of this article will be made available by the authors, without undue reservation.
